# Microbiological Profiles after Out-of-Hospital Cardiac Arrest: Exploring the Relationship between Infection, Inflammation, and the Potential Effects of Mechanical Circulatory Support

**DOI:** 10.3390/jcm13154297

**Published:** 2024-07-23

**Authors:** Julian Kreutz, Charlotte Müller, Georgios Chatzis, Styliani Syntila, Maryana Choukeir, Ann-Christin Schäfer, Susanne Betz, Bernhard Schieffer, Nikolaos Patsalis, Birgit Markus

**Affiliations:** 1 Department of Cardiology, Angiology, and Intensive Care Medicine, University Hospital, Philipps University of Marburg, 35043 Marburg, Germanybirgit.markus@staff.uni-marburg.de (B.M.); 2 Center for Emergency Medicine, University Hospital, Philipps University of Marburg, 35043 Marburg, Germany

**Keywords:** out-of-hospital cardiac arrest (OHCA), post-resuscitation management, mechanical circulatory support, microbiological profile, inflammation, infection

## Abstract

**Background:** Cardiogenic shock (CS) following an out-of-hospital cardiac arrest (OHCA) poses significant management challenges, exacerbated by inflammatory responses and infectious complications. This study investigates the microbiological profiles and impacts of mechanical circulatory support (MCS) on inflammation and infection in OHCA patients. **Methods:** We retrospectively analyzed microbiological data from various specimens of 372 OHCA patients, who were treated at the Cardiac Arrest Center of the University Hospital of Marburg from January 2018 to December 2022. Clinical outcomes were evaluated to investigate the potential impact of MCS on infection and inflammation. **Results:** Of the study cohort, 115 patients received MCS. The microbiological analysis revealed a higher incidence of positive blood cultures in the MCS group vs. the non-MCS group (39% vs. 27.7%, *p* = 0.037), with predominantly Gram-positive bacteria. Patients with positive microbiological findings had longer in-hospital stays and prolonged periods of mechanical ventilation. The levels of inflammatory markers such as C-reactive protein (CRP) and procalcitonin (PCT) differed, suggesting a more pronounced inflammatory response in MCS patients, especially in the later ICU stages. Notably, despite the higher infection rate in the MCS group, the survival rates did not significantly differ in the two groups. **Conclusions:** MCS appears to influence the microbiological and inflammatory landscape in OHCA patients, increasing the susceptibility to certain infections but not affecting the overall mortality. This study underscores the complexity of managing post-resuscitation care and highlights the need for tailored therapeutic strategies to effectively mitigate infectious and inflammatory complications.

## 1. Introduction

The treatment of a broad spectrum of inflammatory conditions and their complications is a major challenge in the therapeutic management of patients with cardiogenic shock (CS) and particularly post-cardiac-arrest shock. This is even more evident when considering that, despite major advances in pre-hospital and in-hospital management, the outcomes after OHCA remain limited, emphasizing the clinical importance of an appropriate and time-critical therapeutic approach [1].

Various inflammation-associated factors and processes seem to be relevant in this context. One of these is post-cardiac-arrest syndrome (PCAS), which, among other factors, results from the reperfusion injury of the ischemic tissue, oxidative stress, and multiple organ failure and significantly worsens the outcomes [2,3]. In addition, the effect of mechanical circulatory support (MCS) on inflammation is controversially discussed in the current literature [4,5,6,7,8,9,10,11,12,13]. However, the use of MCS is often essential to stabilize the hemodynamics in CS after OHCA, including extracorporeal life support (ECLS).

Next to intrinsically triggered systemic inflammatory processes that occur in the context of shock, infectious diseases and their complications must also be considered. In particular, due to the physiological and immunological disturbances and dysregulated innate immune response that occur after cardiopulmonary resuscitation (CPR), patients admitted to the intensive care unit (ICU) often develop infectious complications, such as pneumonia [8,14,15,16,17]. Moreover, factors such as the loss of airway protection, the induction of coma, the need for emergency airway access, and mechanical ventilation further increase the risk of early-onset infections, predisposing patients to complications. The use of MCS can also lead to an increased rate of infections, as large bore venous and arterial accesses often need to be placed quickly in an emergency situation [18].

To monitor inflammation and infection, the levels of several biomarkers, such as C-reactive protein (CRP), procalcitonin (PCT), and interleukin-6 (IL-6), appear to be associated with the presence of inflammatory processes and the severity of CS, not only in plasma but also in serous body fluids such as pulmonary edema [3]. Ultimately, due to the different therapeutic approaches, a distinction must be made between systemic inflammatory response syndrome (SIRS), which is caused by pathogenic microorganisms, and sepsis-like conditions such as PCAS. The delayed diagnosis of infectious complications leads to the late initiation of anti-microbiological treatment, resulting in the prolongation of ICU and hospital stays, the delayed initiation of rehabilitation and reintegration, and other consequences, increasing, in turn, the economic burden on healthcare systems [19].

Here, we performed a comprehensive analysis of the microbiological profiles of OHCA patients, while evaluating the role of MCS on inflammation and infectious complications during therapy. Thus, we aimed to refine the diagnostic and therapeutic approaches, potentially improving patient outcomes by facilitating the timely initiation of appropriate therapy.

## 2. Materials and Methods

### 2.1. Patient Cohort and Study Design

This single-center, retrospective analysis examined real-world patient data from individuals admitted to the Cardiac Arrest Center (CAC) at the University Hospital of Marburg after OHCA between January 2018 and December 2022. The inclusion criteria were age ≥ 18 years, non-traumatic cardiac arrest, and pre-hospital resuscitation by the emergency medical services (EMS). Patients who died in the emergency department and patients who did not undergo microbiologic testing during ICU care were excluded from this study. The study included an evaluation of documented pre-hospital data, as well as inpatient data from the hospital’s internal databases. In detail, all blood cultures, urine cultures, tracheal secretions, bronchoalveolar lavages (BAL), and puncture specimens (either ascites or pleural fluid) collected during the in-hospital stay at the ICU were analyzed for their microbiological results and spectra. To ensure the clinical relevance of our findings, only those pathogens found in the respective sample type, which were identified in at least two patients or more within the cohort, are reported and analyzed (Tables 3–7).

The criteria for the implantation of MCS included hemodynamic instability with the need for inotropes and vasopressors to maintain a mean arterial blood pressure of about ≥65 mmHg, severely depressed left ventricular function, and evidence of hypoperfusion or organ failure. Impella devices were used for primary left ventricular dysfunction when oxygenation was adequate. VA-ECMO was indicated for biventricular failure, a Horowitz index of <200 mmHg, and during ongoing resuscitation if the criteria for extracorporeal CPR (eCPR) were met (witnessed cardiovascular arrest, minimal no-flow time, age <75, no major comorbidities or malignancy). Well-known scoring systems were used to assess the severity of shock. The revised post-Cardiac Arrest Syndrome for Therapeutic hypothermia (rCAST) score was calculated using complete data sets, including the initial arrest rhythm, time to ROSC, arterial pH, lactate levels, and Glasgow Coma Scale Motor Score [20]. In addition, the Society for Cardiovascular Angiography and Interventions (SCAI) classification system was used to categorize the severity of CS according to the underlying definitions [21,22].

### 2.2. Statistical Analysis

Statistical analyses were performed using IBM SPSS Statistics version 29 and GraphPad Prism version 10. Continuous variables were summarized as the mean ± SD, and differences between the survivor and non-survivor groups were tested with independent-samples t-tests using Satterthwaite’s adjustment for unequal variances. Categorical variables were analyzed using the chi-squared test (χ^2^) with N-1 adjustment, whereas nonparametric data were analyzed using the Mann–Whitney U test. One-way ANOVA was used to compare three or more groups. All statistical analyses were performed at a predetermined significance level of 0.05. Pearson’s biserial correlation was used to assess the linear relationships between variables, with correlation coefficients near +1 or −1 indicating strong positive or negative relationships. In addition, Spearman’s nonparametric rank correlation coefficient was used to assess associations between continuous or ordinal variables. To isolate the effects of the main variable of interest on the outcomes, partial nonparametric correlations were performed, controlling for the ICU length of stay. Logistic regression was used to determine the impact of various microbiological findings on patient survival, with the results expressed as odds ratios (OR) with 95% confidence intervals (CI) to elucidate the strength and significance of these associations. In the calculation of correlations and logistic regression, the statistical analyses of each microbiological test (blood culture, urine culture, tracheal secretion, BAL, and punctate) included only those patients who received these tests in the ICU.

### 2.3. Ethics

The local ethics committee of the Philipps University of Marburg approved this retrospective study in accordance with the Declaration of Helsinki (reference ek_mr_14072021).

## 3. Results

### 3.1. Study Cohort

**Figure 1:**
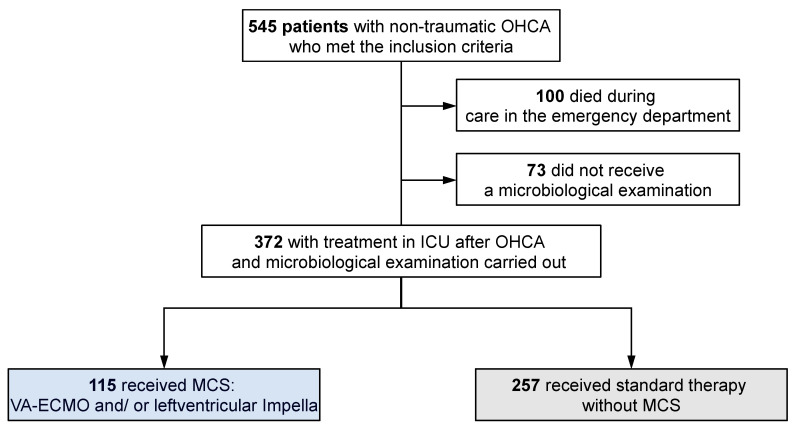
Study cohort. Abbreviations—OHCA: out-of-hospital cardiac arrest; ICU: intensive care unit; MCS: mechanical circulatory support.

A total of 564 patients with OHCA were referred to the CAC at the University Hospital of Marburg during the study period. Of these, eight patients were excluded from this study because they had a traumatic cause of cardiac arrest, four patients had no EMS resuscitation (bystander CPR only), and seven patients had missing data on the resuscitation event. In addition, patients who died during treatment in the emergency department (*n* = 100) and those without microbiological testing (*n* = 73) were excluded from further analysis. Thus, data were analyzed for 372 patients, of whom 115 (31%) received MCS and 257 (69%) patients were treated without MCS support (non-MCS) (Figure 1).

This study included a comprehensive analysis (Table 1) of the demographics and comorbidities across the entire cohort, with a focus on comparing patients based on whether they were treated with MCS (MCS group versus non-MCS group). The analysis revealed that individuals in the MCS group were significantly younger than those in the non-MCS group (58.3 ± 13.1 years vs. 68.0 ± 13.2 years, *p* < 0.001). In addition, the prevalence of comorbidities was significantly lower in the MCS group. This included particularly a second- or third-degree valvular heart disease of the aortic or mitral valve (2.8% vs. 8.7%, *p* = 0.045), pre-existing atrial fibrillation (5.6% vs. 15.4%, *p* = 0.011), arterial hypertension (40.2% vs. 53.9%, *p* = 0.017), diabetes mellitus (10.3% vs. 20.5%, *p* = 0.020), and the need for renal replacement therapy (0% vs. 4.3%, *p* = 0.024). In addition, lower incidences of COPD GOLD stage ≥ 2 (3.7% vs. 11.4%, *p* = 0.021), a history of stroke (3.7% vs. 10.6%, *p* = 0.033), and underlying malignancies (2.8% vs. 10.2%, *p* = 0.018) were observed in the MCS group. The incidence of specific viral infections was minimal, with only six cases of SARS-CoV-2 and two cases of influenza B detected in the study cohort.

Moreover, data regarding the resuscitation event were analyzed for the entire cohort and compared between MCS and non-MCS patients (Table 2). Compared to non-MCS patients, MCS patients were significantly more likely to have an initial shockable rhythm (56.5% vs. 41.2%, *p* = 0.006), to have a longer resuscitation time to the return of spontaneous circulation (ROSC) (47.5 IQR 20.8–94.8 vs. 15.0 IQR 10.0–25.0, *p* < 0.001), and to have been resuscitated with a chest compression device (51.3% vs. 4.3%, *p* < 0.001). A total of 50 patients in the overall cohort (13.4%) underwent eCPR.

After OHCA, most patients exhibited hemodynamic instability during CS, requiring MCS and continuous vasopressor administration. Within the first 24 h of ICU admission, 88.3% of the non-MCS group required vasopressors, compared to 100% of the MCS group. The simultaneous administration of norepinephrine and epinephrine occurred in 20.6% of the non-MCS group, compared to 78.3% of the MCS group (*p* < 0.001). MCS was predominantly implanted on the day of admission in all groups: 96.3% of VA-ECMO patients, 100% of ECMELLA patients (with simultaneous VA-ECMO and Impella implantation in 63.0% and delayed implantation of the second device in 37.0%), and 97.1% of Impella patients.

When comparing the different MCS subgroups within the entire cohort (VA-ECMO *n* = 54; left ventricular Impella *n* = 34; ECMELLA (VA-ECMO and left ventricular Impella) *n* = 27), Impella patients had the highest survival rate (Impella 70.6% vs. VA-ECMO 31.5%/ECMELLA 29.6%; *p* < 0.001). Impella patients had the highest rate of initial shockable rhythm (Impella 85.3% vs. VA-ECMO 35.2%/ ECMELLA 63.0%; *p* < 0.001), while the resuscitation time to ROSC was significantly shorter (25.0 min [IQR 10.0–41.3] vs. VA-ECMO 89.0 min [IQR 30.0–104.0]/ ECMELLA 76.0 min [IQR 25.0–97.0], *p* < 0.001). Additional variables regarding patient characteristics, pre-hospital resuscitation-related parameters, and outcome-relevant parameters in each MCS subgroup are shown in Table 3. There were no significant differences regarding the frequency of positive blood cultures, urine cultures, and tracheal secretions and Gram-positive and Gram-negative pathogens among the MCS groups (Supplementary Table S1).

Of the 372 patients in this study, a total of 357 patients (96.0%) were invasively ventilated, with ventilation initiated preclinically in all cases following OHCA. Of the 15 spontaneously breathing patients, 13 patients were in the non-MCS group and two patients were in the MCS (Impella) group. A total of 341 patients (88.7% in the MCS group and 93.0% in the non-MCS group) underwent whole-body CT (WB-CT) as part of the initial post-resuscitation management in the emergency department. A significant proportion of patients had evidence of pulmonary infiltration and/or aspiration (52.0% in the MCS group and 52.3% in the non-MCS group), and, in some cases, other basal ventilation disorders were diagnosed (18.6% in the MCS group and 21.3% in the non-MCS group).

As a result of these and other findings, a relevant proportion (91.1%) of patients (90.4% MCS patients and 91.3% non-MCS patients) received early antibiotic therapy (within the first 24 h after admission). In the few remaining patients who did not receive early antimicrobial therapy, there was either no evidence of a relevant infection in the presence of spontaneous respiration or the patient died within a very short time (<12 h) in the ICU. We administered ampicillin/sulbactam as an initial empirical therapy followed by calculated regimens (Supplementary Table S2). The frequency of the different antibiotics received during the ICU stay is shown in Supplementary Table S3, with MCS patients receiving different antibiotics more frequently than non-MCS patients (20.0% vs. 5.5%).

### 3.2. Comparison of Patients with Positive and Negative Microbiology Test Results

Regardless of whether patients received MCS, their demographics and preexisting conditions were analyzed in a comparison of patients with positive versus negative microbiological findings (Supplementary Table S4). Microbiological evidence was categorized as follows: 340 patients had blood cultures, of which 106 (31.2%) were positive; 271 patients had urine cultures, of which 69 patients (25.5%) had positive samples. Of 210 patients with tracheal secretion samples, 200 (95.2%) were positive, and of 64 patients with bronchoalveolar lavage (BAL), 27 (42.2%) were positive. In addition, 14 patients had punctuates (ascites/pleura), of whom six (42.9%) were accompanied by positive results. In total, 270 patients (72.6%) exhibited positive microbiology findings in at least one category. The first microbiological examination was performed within the first day of ICU admission in 83.9% of patients. According to our data, patients with positive microbiological findings had a significantly longer in-hospital stay (median 12.0 days [IQR 5.0–19.0] vs. median 7.0 days [IQR 2.0–13.3], *p* < 0.001) and longer duration of mechanical ventilation (median 178.0 h [IQR 75.8–312.0] vs. median 76.5 h [IQR 13.5–180.3], *p* < 0.001).

### 3.3. Comparison of the Microbiology Test Results in the Overall Cohort and MCS vs. Non-MCS Patients

#### 3.3.1. Results from Blood Cultures

Paired aerobic/anaerobic blood cultures were performed in 340 of the total 372 patients, of which 106 patients (31.2%) were positive. The incidence of positive blood cultures was significantly higher in patients with MCS compared to non-MCS patients (39.0% vs. 27.7%, *p* = 0.037). Blood cultures were routinely taken at an early stage after admission to the ICU. Among the patients with positive cultures during their hospital stay, 62.5% had positive findings from samples taken within the first 72 h of ICU treatment.

A comprehensive analysis revealed the predominance of Gram-positive pathogens, which accounted for 87.7% of the positive cultures. Notably, Gram-positive infections were more common in patients with MCS (35.2% vs. 23.8%, *p* = 0.029). Staphylococcus species, followed by Propioni bacteriaceae, Streptococcus species, and Corynebacteriaceae, were the most common. In contrast, the prevalence of Gram-negative pathogens such as Klebsiella species, Escherichia coli, and Proteus mirabilis did not differ significantly between the MCS and non-MCS groups. Anaerobic bacteria were rarely detected, accounting for only 2.4% of the positive cultures. The blood culture results are summarized in Table 4.

#### 3.3.2. Results from Urine Cultures

Urine cultures were obtained from 271 out of 372 patients during their in-hospital stay at the ICU, with 69 patients (25.5%) showing positive results. Notably, a significantly lower prevalence of positive urine cultures was observed in patients with MCS compared to those without MCS (13.9% vs. 29.6%, *p* = 0.009). The microbial spectrum of positive cultures was dominated by Gram-negative pathogens including Escherichia coli, various Gram-negative bacilli, Klebsiella species, and Pseudomonas aeruginosa. Although these Gram-negative pathogens were also more frequently identified in patients without MCS, the differences did not reach statistical significance. Gram-positive pathogens, represented mainly by Enterococcus and Staphylococcus species, represented a smaller proportion of the positive findings, with similar incidence rates observed in both MCS and non-MCS patients. In addition, fungal pathogens, particularly Candida albicans and Candida glabrata, were detected in 4.8% of all positive urine cultures (Table 5).

#### 3.3.3. Results from Tracheal Secretions

The microbiological examination of tracheal secretions was performed in 210 patients of the study cohort. Positive results could be detected in 200 patients (95.2%). Tracheal specimens were found to contain predominantly Gram-positive bacteria, with Streptococcus, Staphylococcus, and Enterococcus being the most common. The analysis revealed no significant difference in the frequency of Gram-positive infections between patients with and without MCS (Table 6).

However, there was a notable difference in the frequency of Gram-negative pathogens, which was significantly lower in MCS patients (37.1%) than in non-MCS patients (56.8%, *p* = 0.010). Gram-negative bacteria included Neisseria, Haemophilus, and Klebsiella species. Fungal colonization was also common in tracheal secretions: 58.6% of patients had positive results in tracheal secretions, primarily displaying Candida albicans, followed by other Candida species and Aspergillus niger.

#### 3.3.4. Results from Bronchoalveolar Lavage (BAL)

During the ICU stay, BAL was performed in 32 patients. Subsequent microbiological analysis revealed the presence of pathogens in 27 of these patients (84.4%). The results indicated a significant prevalence of Gram-positive bacterial and fungal colonization in BAL specimens from patients after OHCA. Among the Gram-positive isolates, Streptococcus species, especially the Viridans group, as well as Staphylococcus and Enterococcus species, were frequently identified. Fungal colonization was predominantly attributed to Candida albicans and other Candida species. The spectrum of Gram-negative bacteria included Neisseria, Klebsiella, and Serratia marcescens. A summary of the results is shown in Table 7.

#### 3.3.5. Results from Pleural or Ascitic Fluid Samples

The pleural or ascitic fluid of 12 patients was subjected to microbiological analysis, showing a 50% positivity rate for the presence of microorganisms. The pathogens detected included Staphylococcus species, Escherichia coli, Klebsiella pneumoniae, and the rare Veillonella atypica. No significant differences were found between MCS and non-MCS patients (Table 8).

### 3.4. Correlation between Microbiological Findings and Length of ICU Stay

To investigate whether the length of the ICU stay was correlated with positive microbiological findings, we performed a point biserial correlation analysis. Overall, we observed a significant positive correlation (Pearson correlation: 0.228; *p* < 0.001). In the individual analyses of the respective microbiological tests, however, the correlations were not significant in each group: blood cultures (Pearson correlation: 0.033; *p* = 0.542), urine cultures (Pearson correlation: −0.024; *p* = 0.694), tracheal secretions (Pearson correlation: −0.010; *p* = 0.883), BAL (Pearson correlation: 0.139; *p* = 0.447), and punctates (Pearson correlation: −0.5010; *p* = 0.097).

### 3.5. Correlation between Microbiological Findings and Survival

Logistic regression analysis was performed to determine whether there were significant associations between hospital survival and the microbiological findings in the overall cohort and in the MCS and non-MCS subgroups. The analysis showed no significant associations between positive blood cultures and survival in the overall cohort (OR 0.647; 95% CI 0.407–1.026; *p* = 0.064), within the MCS group (OR 0.887; 95% CI 0.403–1.951; *p* = 0.766), or within the non-MCS group (OR 0.598; 95% CI 0.335–1.068; *p* = 0.082). In addition, there was no significant difference in the effect of positive blood cultures on survival between the MCS and non-MCS groups (*p* = 0.430). Similarly, positive urine cultures were not significantly associated with survival in the overall cohort (OR 1.020; 95% CI 0.583–1.785; *p* = 0.945), the MCS group (OR 1.500; 95% CI 0.385–5.482; *p* = 0.559), or the non-MCS group (OR 0.843; 95% CI 0.450–1.579; *p* = 0.584). There was also no significant difference in the impact of positive urine cultures on survival between the MCS and non-MCS groups (*p* = 0.451). Furthermore, no significant associations could be detected between positive tracheal secretions and survival in the overall cohort (OR 0.762; 95% CI 0.191–3.038; *p* = 0.700), the MCS group (OR 0.986; 95% CI 0.153–6.367; *p* = 0.988), or the non-MCS group (OR 0.480; 95% CI 0.052–4.408; *p* = 0.516), as well as in the comparison between the MCS and non-MCS groups (*p* = 0.626). Analyses of BAL and punctuates showed extensive confidence intervals due to the small number of cases, which limits the interpretability of these results.

### 3.6. Correlation between the Levels of Inflammatory Markers and Survival

This study evaluated markers of inflammation, specifically CRP and PCT. On day 1, the median CRP levels were significantly lower in the MCS group (3.90 mg/L [IQR 1.40–8.78]) compared to the non-MCS group (6.95 mg/L [IQR 2.43–22.90], *p* = 0.015). This trend continued up to day 3, with the MCS group showing a median CRP of 124.80 mg/L (IQR 95.08–163.60) versus 139.90 mg/L (IQR 101.20–189.20) in the non-MCS group (*p* = 0.033). However, by day 7, this situation was reversed, with the MCS group showing significantly higher median CRP levels (167.50 mg/L [IQR 113.40–232.30]) compared to the non-MCS group (126.10 mg/L [IQR 73.15–180.70], *p* < 0.001).

**Figure 2:**
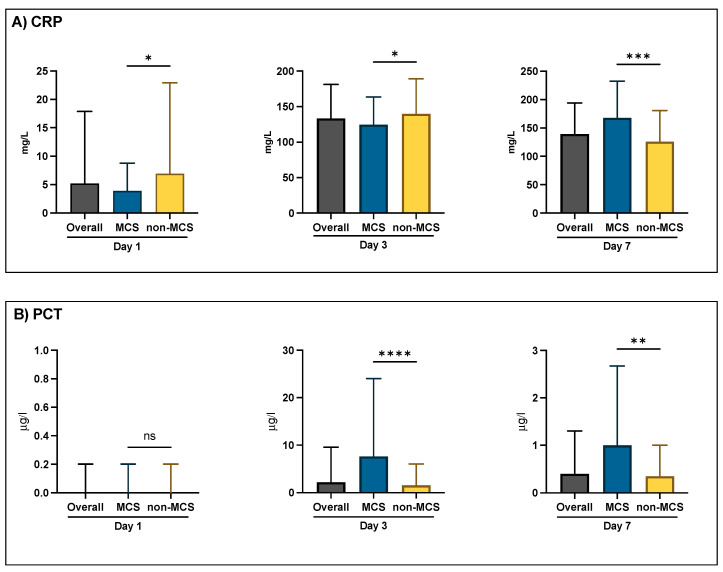
Levels of inflammatory biomarkers (CRP and PCT) during the first 7 days of ICU treatment. Abbreviations—MCS: mechanical circulatory support; CRP: C-reactive protein; PCT: procalcitonin. *: *p* < 0.05; **: *p* < 0.01, ***: *p* < 0.001; ****: *p* < 0.0001.; ns: not significant.

The levels of PCT on day 1 showed negligible differences between the groups. However, on day 3, a significant increase in the median PCT levels was observed in the MCS group (7.60 µg/L [IQR 2.20–24.00]) compared to the non-MCS group (1.50 µg/L [IQR 0.40–6.00], *p* < 0. 001), a pattern that persisted through to day 7, with the MCS group having a median PCT of 1.00 µg/L (IQR 0.40–2.68) versus 0.35 µg/L (IQR 0.20–1.00) in the non-MCS group (*p* = 0.005). These results suggest a more pronounced inflammatory response in the MCS group, particularly in the later stages of the ICU stay (Figure 2).

Furthermore, we analyzed the correlations between the CRP and PCT levels on days 1, 3, and 7 and the microbiological findings using Spearman correlations. Only patients who underwent microbiological testing in the appropriate category were included. We used both nonparametric Spearman correlations and partial nonparametric correlations adjusted for the length of the ICU stay. No meaningful differences were found between the adjusted and unadjusted correlations. Significant findings included correlations of CRP with positive urine cultures on day 1 (rho = 0.153; *p* = 0.009) and day 7 (rho = −0.185; *p* = 0.009) and of PCT with blood cultures on day 3 (rho = 0.142; *p* = 0.017). Moreover, CRP and PCT showed significant correlations with tracheal secretions on day 1 (rho = 0.146; *p* = 0.035).

### 3.7. Correlation between SCAI Stage, rCAST Score and Microbiological Findings

Overall, the survival rate in the total cohort decreased with the increasing SCAI stage. In patients with SCAI stage D or E, a higher survival rate was observed in the MCS group compared to the non-MCS group (stage D: 61.9% vs. 47.5%, *p* = 0.117 and stage E: 31.5% vs. 26.3%, *p* = 0.570) (Supplementary Table S5). There was no significant association between the SCAI stage and the percentage of positive microbiological findings taken within the first 24 h of ICU admission, either in the overall cohort (*p* = 0.175) or for MCS (*p* = 0.455) or non-MCS patients (*p* = 0.211) (Supplementary Table S6).

In addition, the rCAST score was obtained within the first 24 h of ICU treatment for patients with complete data (*n* = 366). The mean score was significantly lower in the survivors than in the non-survivors (8.97 ± 3.71 vs. 12.04 ± 3.53, *p* < 0.001). In addition, the rCAST score was higher in the MCS group than in the non-MCS group (12.38 ± 4.01 vs. 9.49 ± 3.56, *p* < 0.001). When evaluating the relationship between the rCAST score and positive microbiological findings, there were no relevant associations in the overall cohort (*p* = 0.849) or in the MCS (*p* = 0.249) and the non-MCS group (*p* = 0.327).

## 4. Discussion

This study investigated the microbiological landscape after non-traumatic OHCA in patients with and without MCS while focusing on individual profiles, inflammatory parameters, and their correlations with the clinical outcomes. We performed a detailed analysis of microbiological data, categorizing microorganisms by compartment while identifying a significantly higher incidence of positive urine and blood cultures in patients with MCS compared to non-MCS patients. Despite this higher incidence, no effect on mortality was observed. This is consistent with the current literature and suggests that although MCS patients appear to be more susceptible to bacteremia, these infections do not necessarily lead to increased mortality in this predisposed patient cohort—for example, if an appropriate antimicrobial treatment regimen is chosen early [17,18,23]. In our and other OHCA cohorts, in particular, Gram-positive Staphylococcus species were detected. Moreover, these bacteria were found significantly more frequently in the MCS group than in the non-MCS group [24].

Regarding the potential role of MCS in the context of infection and inflammation, it has been hypothesized for a long time that VA-ECMO may trigger an inflammatory cascade due to blood contact with the foreign surfaces of an extracorporeal circuit [12]. In contrast, myocardial unloading with the Impella microaxial pump has been shown to have a protective effect on the inflammatory responses of patients [25].

On the other hand, for the first time, Diakos et al. recently described a protective effect of MCS with both Impella and ECMO on inflammation and immunomodulation that appears to overcome even the potential negative effects of extracorporeal circulation, whereas infectious complications are reported to be more prominent in patients receiving any form of VA-ECMO therapy [11,18]. Interestingly, the downregulation of several inflammatory pathways, including regulatory cytokines (NF-κB, CxCl3) involved in monocyte regulation and activation, seems to be device-specific [11]. Moreover, since inflammation has been described as a key factor associated with acute and chronic heart failure, these results may further support the hypothesis of the MCS-induced optimization of myocardial recovery and regeneration [26,27,28].

Our data indicate that despite a longer duration of therapy and increased mechanical ventilation in critically ill patients with Impella, the survival rates were significantly higher compared to other MCS subgroups (ECMELLA and VA-ECMO). Since these conditions should increase the infection rates and mortality, our results support those of Diakos et al., suggesting a protective effect of the Impella device that even overcomes infectious complications. Moreover, this is in accordance with the results of other studies that have also failed to observe a relevant effect of infectious complications on mortality in patients with MCS [18,29]. In our study, elevated PCT levels on day 3 of hospitalization correlated with positive blood cultures, with significant differences between the MCS and non-MCS groups. Thus, at this point, device-associated infections appear to play a critical role. PCT may be an important biomarker for management and decision-making in situations of clinically relevant infections. Early elevated CRP was more likely correlated with positive microbiological results in urine or tracheal secretions, whereas it remains unclear at this very early time point of treatment whether this is inflammation, a relevant infection, or even microbial colonization.

Due to the retrospective design of this study, a more precise distinction between sterile inflammation and infection, based on the regulation of biomarkers, was not possible, as we did not investigate other cytokine, chemokine, or protein levels reflecting the inflammatory status in MCS and non-MCS patients. However, according to our and other data, the focus in patients with and without MCS after OHCA should be on the early prevention of infectious complications, as the activation of anti-inflammatory cascades and signaling pathways during ongoing MCS support appears to promote rather than worsen myocardial recovery and thus may protect organ function. This includes the early monitoring of the CRP and PCT values and the initiation of anti-microbiological treatment. This is also important since some antibiotics are said to have an anti-inflammatory effect, which also supports the need for the rapid initiation of antimicrobial therapy, although the debate continues about the appropriate timing of initiation and the most optimal agents [19,30,31,32]. Furthermore, under the cautious assumption that patients with MCS may be more severely ill than those without MCS, and that this and other factors may potentially lead to a higher number of complications during hospitalization, these negative aspects seem to play a rather negligible role during treatment with MCS, as the mortality did not differ significantly between the two subgroups (MCS vs. non-MCS).

However, the process of microbiological diagnostics extends over a long period in everyday clinical practice. This often necessitates early empirical broad-spectrum antibiotic treatment, which leads to antimicrobial resistance and a lack of response to antibiotic therapy with subsequent complications.

Our findings underscore the importance of integrating early point-of-care testing (POCT) in the ICU for the timely and accurate diagnosis of infections. POCT improves the detection of infectious agents and facilitates prompt and tailored treatment. This approach helps to combat antimicrobial resistance by minimizing the use of broad-spectrum antibiotics, potentially shortening ICU stays, and improving patient outcomes through more targeted therapy.

## 5. Limitations

There are several limitations of our study, which have to be acknowledged. The single-center, retrospective design may limit the generalizability of our findings. Moreover, the exclusion of patients without complete microbiological profiles may have introduced selection bias, potentially affecting the severity and outcomes assessed. In addition, the small sample sizes in certain analyses may have led to underpowered statistical conclusions. Therefore, the reported results should be considered preliminary and we emphasize the need for larger studies to further confirm these findings. The observational nature of this study also makes it difficult to establish causal relationships between the microbiological findings and clinical outcomes. Future research would benefit from a multicenter, prospective design to increase the validity and generalizability.

## 6. Conclusions

In patients with post-cardiac-arrest shock after OHCA, complex interactions between infection and inflammation can be observed. This appears even more relevant when MCS is used. However, despite the increased incidence of positive microbiological findings in MCS patients, the survival rate remained unchanged compared to the non-MCS group. This suggests that the increased infection rate associated with MCS is less of a problem in terms of outcomes. This could be due to effective early antimicrobial diagnostics and intervention, but apparently also due to the MCS systems themselves, as these appear to alter individual inflammatory profiles and responses. To improve the outcomes, future research now needs to further open the “Pandora’s Box” of infection and inflammation during MCS support after OHCA, enabling the development of the optimal diagnostic pathways and targeted therapeutic strategies for post-resuscitation management.

## Figures and Tables

**Table 1:** Demographics and comorbidities of the overall cohort and MCS vs. non-MCS patients. Abbreviations—*n*: number of patients with valid data; BMI: body mass index; MI: myocardial infarction; CHD: coronary heart disease; py: pack years; COPD: chronic obstructive pulmonary disease; OSAS: obstructive sleep apnea syndrome; PAE: pulmonary artery embolism. ^1^: *n* (%); ^2^: Mean (SD).

	*n*=	Overall Cohort	MCS	Non-MCS	*p*-Value
Number of patients ^1^		372	115 (30.9)	257 (69.1)	
Age (years) ^2^	372	65.0 (±13.9)	58.3 (±13.1)	68.0 (±13.2)	<0.001
Male sex ^1^	372	278 (74.7)	91 (79.1)	187 (72.8)	0.192
BMI (kg/m^2^) ^2^	266	28.4 (±5.8)	29.2 (±7.1)	27.9 (±5.0)	0.119
MI in the past/CHD ^1^	361	59 (15.9)	14 (13.1)	45 (17.7)	0.277
Vitium of aortic/mitral valve (grade 2/3) ^1^	361	25 (6.7)	3 (2.8)	22 (8.7)	0.045
Heart failure ≥ NYHA 3 ^1^	361	24 (6.5)	4 (3.7)	20 (7.9)	0.150
Atrial fibrillation ^1^	361	45 (12.1)	6 (5.6)	39 (15.4)	0.011
Pacemaker ^1^	361	12 (3.2)	3 (2.8)	9 (3.5)	0.720
Arterial hypertension ^1^	361	180 (4.8)	43 (40.2)	137 (53.9)	0.017
Hyperlipidemia ^1^	361	57 (15.3)	11 (10.3)	46 (18.1)	0.062
Diabetes mellitus ^1^	361	63 (16.9)	11 (10.3)	52 (20.5)	0.020
Nicotine abuse (>5py) ^1^	361	91 (24.5)	27 (25.2)	64 (25.2)	0.994
Alcohol abuse ^1^	361	25 (6.7)	7 (6.5)	18 (7.1)	0.852
Chronic renal failure KDIGO ≥ stage 3 ^1^	369	42 (11.3)	8 (7.0)	34 (13.4)	0.072
Renal replacement therapy ^1^	369	11 (3.0)	0	11 (4.3)	0.024
COPD ≥ GOLD 2 ^1^	361	33 (8.9)	4 (3.7)	29 (11.4)	0.021
Bronchial asthma ^1^	361	11 (3.0)	5 (4.7)	6 (2.4)	0.243
OSAS ^1^	361	14 (3.8)	3 (2.8)	11 (4.3)	0.493
Apoplexy ^1^	361	41 (11.0)	4 (3.7)	37 (10.6)	0.033
Thrombosis/PAE ^1^	361	9 (2.4)	3 (2.8)	6 (2.4)	0.806
Malignant disease ^1^	361	29 (7.8)	3 (2.8)	26 (10.2)	0.018
Peripheral arterial disease ≥ stage 2 ^1^	361	14 (3.8)	4 (3.7)	10 (3.9)	0.929
Carotid artery stenosis ^1^	361	13 (3.5)	3 (2.8)	10 (3.9)	0.598
Hypo- or hyperthyroidism ^1^	361	27 (7.3)	7 (6.5)	20 (7.9)	0.660

**Table 2:** Pre-hospital resuscitation-associated parameters of the overall cohort and MCS vs. non-MCS patients. Abbreviations—*n*: number of patients with valid data; VF: ventricular fibrillation; VT: ventricular tachycardia; CPR: cardiopulmonary resuscitation; ROSC: return of spontaneous circulation. ^1^: *n* (%); ^3^: Median (IQR).

	Overall Cohort *n* = 372	MCS *n* = 115	Non-MCS *n* = 257	*p*-Value
Initial shockable rhythm (VF/VT) ^1^	171 (46.0)	65 (56.5)	106 (41.2)	0.006
Witnessed cardiac arrest ^1^	259 (69.6)	77 (67.0)	182 (70.8)	0.454
Performed bystander CPR ^1^	224 (60.2)	71 (61.7)	153 (59.5)	0.688
Resuscitation time until ROSC ^3^	20.0 (10.0–35.0)	47.5 (20.8–94.8)	15.0 (10.0–25.0)	<0.001
Mechanical CPR (chest compression device) ^1^	70 (18.8)	59 (51.3)	11 (4.3)	<0.001

**Table 3:** Different MCS subgroups (VA-ECMO, ECMELLA (VA-ECMO + Impella) and Impella). ^1^: *n* (%); ^2^: Mean (SD), ^3^: Median (IQR).

	VA-ECMO	ECMELLA	Impella	*p*-Value
Number of patients	54	27	34	
Age (years) ^2^	56.7 (±13.5)	56.4 (±12.2)	62.3 (12.6)	0.212
Initial shockable rhythm (VF/VT) ^1^	19 (35.2)	17 (63.0)	29 (85.3)	<0.001
Witnessed cardiac arrest ^1^	38 (70.4)	17 (63.0)	22 (64.7)	0.787
Performed bystander CPR ^1^	33 (61.1)	15 (55.6)	23 (67.6)	0.640
Resuscitation time until ROSC ^3^	89.0 (30.0–104.0)	76.0 (25.0–97.0)	25.0 (10.0–41.3)	<0.001
Mechanical CPR (chest compression device) ^1^	36 (66.7)	18 (66.7)	5 (14.7)	<0.001
Duration of in-hospital stay (days) ^3^	3.0 (1.0–16.3)	6.0 (2.0–22.0)	17.5 (10.0–22.3)	0.001
Mechanical ventilation (hours) ^3^	64.0 (16.8–277.5)	159.0 (40.0–455.0)	239.0 (155.5–402.0)	0.047
Duration of MCS (days), survivors ^3^	9.0 (8.0–13.0)	9.5 (7.0–15.5)	6.0 (5.0–8.0)	0.003
Duration of MCS (days), non-survivors ^3^	2.0 (1.5–4.5)	4.0 (2.0–8.0)	5.5 (1.8–11.0)	0.032
Survived ^1^	17 (31.5)	8 (29.6)	24 (70.6)	<0.001

**Table 4:** Blood culture analysis in the overall cohort and MCS vs. non-MCS patients. Abbreviations—*n*: number of patients with valid data; MRSA: methicillin-resistant Staphylococcus aureus; 3MRGN: isolates resistant to 3 out of 4 relevant antimicrobial classes (acylureidopenicillin, 3rd/4th generation cephalosporins, carbapenems, fluoroquinolones). ^1^: *n* (%).

	Overall	MCS	Non-MCS	*p*-Value
Patients with blood culture (*n*=)	340	105	235	
Positive samples ^1^	106 (31.2)	41 (39.0)	65 (27.7)	0.037
**Gram-positive pathogens ^1^**	93 (27.4)	37 (35.2)	56 (23.8)	0.029
Staphylococcus species ^1^	76 (22.4)	27 (25.7)	49 (20.9)	0.321
Staphylococcus epidermidis ^1^	54 (15.9)	22 (21.0)	32 (13.6)	0.088
Staphylococcus aureus (including MRSA) ^1^	2 (0.6)	0 (0.0)	2 (0.9)	0.344
Other staphylococci ^1^	29 (8.5)	7 (6.7)	22 (9.4)	0.412
Propionibacteriacae ^1^	14 (4.1)	7 (6.7)	7 (3.0)	0.114
Streptococcus species ^1^	5 (1.5)	3 (2.9)	2 (0.9)	0.156
Corynebacteriacae ^1^	3 (0.9)	1 (1.0)	2 (0.9)	0.927
**Gram-negative pathogens ^1^**	12 (3.5)	2 (1.9)	10 (4.3)	0.279
Klebsiella species ^1^	5 (1.5)	1 (1.0)	4 (1.7)	0.596
Escherichia coli (including 3MRGN) ^1^	4 (1.2)	1 (1.0)	3 (1.3)	0.798
Proteus mirabilis ^1^	2 (0.6)	/	2 (0.9)	0.344
**Anaerobic pathogens ^1^**	8 (2.4)	5 (4.8)	3 (1.3)	0.050
Bacteroides species ^1^	2 (0.6)	1 (1.0)	1 (0.4)	0.523

**Table 5:** Urine culture analysis results in the overall cohort and MCS vs. non-MCS patients. Abbreviations—*n*: number of patients with valid data; 3MRGN: isolates resistant to 3 out of 4 relevant antimicrobial classes (acylureidopenicillin, 3rd/4th generation cephalosporins, carbapenems, fluoroquinolones). ^1^: *n* (%).

	Overall Cohort	MCS	Non-MCS	*p*-Value
Patients with urine culture (*n*=)	271	72	199	
Positive samples ^1^	69 (25.5)	10 (13.9)	59 (29.6)	0.009
**Gram-positive pathogens ^1^**	23 (8.5)	3 (4.2)	20 (10.1)	0.125
Enterococcus species ^1^	15 (5.5)	2 (2.8)	13 (6.5)	0.233
Staphylococcus species ^1^	6 (2.2)	1 (1.4)	5 (2.5)	0.579
**Gram-negative pathogens ^1^**	45 (16.6)	7 (9.7)	38 (19.1)	0.068
Escherichia coli (including 3MRGN) ^1^	21 (7.7)	3 (4.2)	18 (9.0)	0.185
Gram-negative rods ^1^	17 (6.3)	4 (5.6)	13 (6.5)	0.770
Klebsiella species ^1^	4 (1.5)	/	4 (2.0)	0.226
Pseudomonas aeruginosa (including 3MRGN) ^1^	4 (1.5)	/	4 (2.0)	0.226
**Mycosis ^1^**	13 (4.8)	1 (1.4)	12 (6.0)	0.115
Candida species ^1^	7 (2.6)	/	7 (3.5)	0.108

**Table 6:** Analysis of tracheal secretions in the overall cohort and MCS vs. non-MCS patients. Abbreviations—*n*: number of patients with valid data. ^1^: *n* (%).

	Overall Cohort	MCS	Non-MCS	*p*-Value
Patients with tracheal secretion (*n*=)	210	62	148	
Positive samples ^1^	200 (95.2)	57 (91.9)	143 (96.6)	0.147
**Gram-positive pathogens ^1^**	161 (76.7)	47 (75.8)	114 (77.0)	0.849
Streptococcus species ^1^	135 (64.3)	40 (64.5)	95 (64.2)	0.964
Staphylococcus species ^1^	73 (34.8)	16 (25.8)	57 (38.5)	0.078
Enterococcus species ^1^	41 (19.5)	15 (24.2)	26 (17.6)	0.270
**Gram-negative pathogens ^1^**	107 (51.0)	23 (37.1)	84 (56.8)	0.010
Neisseria species ^1^	44 (21.0)	9 (14.5)	35 (23.6)	0.139
Haemophilus species ^1^	26 (12.4)	7 (11.3)	19 (12.8)	0.757
Klebsiella species ^1^	21 (10.0)	1 (1.6)	20 (13.5)	0.009
**Mycosis ^1^**	123 (58.6)	33 (53.2)	90 (60.8)	0.310
Candida species	41 (19.5)	8 (12.9)	33 (22.3)	0.118
Candida albicans	103 (49.0)	28 (45.2)	75 (50.7)	0.467
Candida glabrata	2 (1.0)	1 (1.6)	1 (0.7)	0.525
Aspergillus niger	2 (1.0)	/	2 (1.4)	0.359

**Table 7:** Analysis of bronchoalveolar lavage (BAL) in the overall cohort and MCS vs. non-MCS patients. Abbreviations—*n*: number of patients with valid data. ^1^: *n* (%).

	Overall Cohort	MCS	Non-MCS	*p*-Value
Patients with BAL (*n*=)	32	11	21	
Positive samples ^1^	27 (84.4)	8 (72.7)	19 (90.5%)	0.196
**Gram-positive pathogens ^1^**	17 (53.1)	6 (54.5)	11 (52.4)	0.909
Streptococcus species ^1^	11 (34.4)	4 (36.4)	7 (33.3)	0.866
Staphylococcus species ^1^	9 (28.1)	2 (18.2)	7 (33.3)	0.373
Enterococcus species ^1^	5 (15.6)	3 (27.3)	2 (9.5)	0.196
**Gram-negative pathogens**	8 (25.0)	1 (9.1)	7 (33.3)	0.139
Neisseria species ^1^	3 (9.4)	1 (9.1)	2 (9.5)	0.969
Klebsiella species ^1^	2 (6.3)	/	2 (9.5)	0.298
**Mycosis ^1^**	17 (53.1)	6 (54.5)	11 (52.4)	0.909
Candida species ^1^	5 (15.6)	/	5 (23.8)	0.083
Candida albicans ^1^	14 (43.8)	6 (54.5)	8 (38.1)	0.381

**Table 8:** Analysis of pleural or ascitic fluid samples in the overall cohort and in MCS vs. non-MCS patients. Abbreviations—*n*: number of patients with valid data. ^1^: *n* (%).

	Overall Cohort	MCS	Non-MCS	*p*-Value
Patients with pleural/ascitic fluid samples (*n*=)	12	5	7	
Positive samples ^1^	6 (50.0)	3 (60.0)	3 (42.9)	0.575
**Gram-positive pathogens ^1^**	3 (25.0)	2 (40.0)	1 (14.3)	0.322
Staphylococcus species ^1^	3 (25.0)	2 (40.0)	1 (14.3)	0.322
**Gram-negative pathogens ^1^**	2 (16.6)	1 (20.0)	1 (14.3)	0.802

## Data Availability

The data presented in this study are available upon request from the corresponding author. The data are not publicly available for ethical reasons.

## Supplementary Materials

The following supporting information can be downloaded at: https://www.mdpi.com/article/10.3390/jcm13154297/s1, Table S1: Microbiological findings in the different MCS subgroups; Table S2: Frequency of different antibiotics during ICU treatment for the overall cohort and separately for the MCS group and the non-MCS group; Table S3: Number of different antibiotics used during ICU treatment for the overall cohort and separately for the MCS group and the non-MCS group; Table S4: Demographics and pre-existing conditions of patients with positive and negative microbiology test results; Table S5: Survival rates per SCAI stage of cardiogenic shock (A–E) for the overall cohort and separately for the MCS group and the non-MCS group; Table S6: Frequency of positive microbiological findings per SCAI stage of cardiogenic shock (A–E) in the overall cohort and in the MCS group and the non-MCS group.
