# Supplementary material for: Microbiological Profiles after Out-of-Hospital Cardiac Arrest: Exploring the Relationship between Infection, Inflammation, and the Potential Effects of Mechanical Circulatory Support
Source: J Clin Med. 2024 Jul 23;13(15):4297. doi: 10.3390/jcm13154297 (PMC11312496; doi:10.3390/jcm13154297)
Supplement: Supplementary file 1 [file jcm-13-04297-s001.zip › jcm-3103173-supplementary.pdf]

Supplementary Table S1. Microbiological findings in the different MCS subgroups (VA-ECMO, ECMELLA (VA-ECMO + Impella) and Impella). [1]: *n* (%).

	**VA-ECMO** *n*=54	**ECMELLA** *n*=27	**Impella** *n*=34	***p*-value**
**Patients with blood culture (*n*=)**	49	25	31	
Patients with positive samples[1]	18 (36.7)	10 (40.0)	13 (41.9)	0.903
Gram-positive pathogens[1]	16 (32.7)	9 (36.0)	12 (38.7)	0.867
Gram-negative pathogens[1]	1 (2.0)	0 (0.0)	1 (3.2)	1.000
**Patients with urine culture (*n*=)**	28	19	25	
Patients with positive samples[1]	5 (17.9)	2 (10.5)	3 (12.0)	0.757
Gram-positive pathogens[1]	3 (10.7)	0 (0.0)	0 (0.0)	0.110
Gram-negative pathogens[1]	3 (10.7)	1 (5.3)	3 (12.0)	0.785
**Patients with tracheal secretion (*n*=)**	23	16	23	
Patients with positive samples[1]	20 (87.0)	16 (100)	21 (91.3)	0.424
Gram-positive pathogens[1]	17 (73.9)	14 (87.5)	16 (69.6)	0.485
Gram-negative pathogens[1]	9 (39.1)	6 (37.5)	8 (34.8)	1.000

Supplementary Table S2. Frequency of different antibiotics during ICU treatment for the overall cohort and separately for the MCS group and the non-MCS group. [1]: *n* (%).

	**Overall cohort** *n*= 372	**MCS** *n*=115	**Non-MCS** *n*=257	***p*-value**
**Amoxicillin**[1]	2 (0.5)	0 (0.0)	2 (0.8)	0.572
**Ampicillin/Sulbactam**[1]	213 (57.3)	60 (52.2)	153 (59.5)	0.212
**Azithromycin**[1]	3 (0.8)	1 (0.9)	2 (0.8)	1.000
**Ceftazidime**[1]	19 (5.1)	13 (11.3)	6 (2.3)	**<0.001**
**Ceftriaxone**[1]	7 (1.9)	1 (0.9)	6 (2.3)	0.444
**Ciprofloxacin**[1]	2 (0.5)	0 (0.0)	2 (0.8)	0.572
**Clarithromycin**[1]	72 (19.4)	21 (18.3)	51 (19.8)	0.778
**Clindamycin**[1]	12 (3.2)	4 (3.5)	8 (3.1)	1.000
**Cotrimoxazol**[1]	1 (0.3)	0 (0.0)	1 (0.4)	1.000
**Daptomycin**[1]	1 (0.3)	1 (0.9)	0 (0.0)	0.309
**Flucloxacillin**[1]	3 (0.8)	2 (1.7)	1 (0.4)	0.227
**Fosfomycin**[1]	21 (5.6)	13 (11.3)	8 (3.1)	**0.003**
**Gentamicin**[1]	1 (0.3)	0 (0.0)	1 (0.4)	1.000
**Levofloxacin**[1]	133 (35.8)	48 (41.7)	85 (33.1)	0.128
**Linezolid**[1]	24 (6.5)	10 (8.7)	14 (5.4)	0.257
**Meropenem**[1]	45 (12.1)	27 (23.5)	18 (7.0)	**<0.001**
**Moxifloxacin**[1]	1 (0.3)	1 (0.9)	0 (0.0)	0.309

**Piperacillin/Tazobactam**[1]	218 (58.6)	70 (60.9)	148 (57.6)	0.571
**Rifampicin**[1]	1 (0.3)	0 (0.0)	1 (0.4)	1.000
**Teicoplanin**[1]	1 (0.3)	0 (0.0)	1 (0.4)	1.000
**Vancomycin**[1]	63 (16.9)	33 (28.7)	30 (11.7)	**<0.001**

Supplementary Table S3. The number of different antibiotics used during ICU treatment for the overall cohort and separately for the MCS group and the non-MCS group. [1]: *n* (%).

**Number of different antibiotics** during ICU stay	***n*=**	**Overall cohort**	**MCS**	**Non-MCS**
no antibiotics[1]	369	33 (8.9)	11 (9.6)	22 (8.6)
1-2 antibiotics[1]	369	177 (48.0)	46 (40.0)	131 (51.6)
3-4 antibiotics[1]	369	122 (33.1)	35 (30.4)	87 (34.3)
>5 antibiotics[1]	369	37 (10.0)	23 (20.0)	14 (5.5)

Supplementary Table S4. Demographics and pre-existing conditions of patients with positive and negative microbiology test results. Abbreviations: MI: myocardial infarction; CHD: coronary heart disease; py: pack years; COPD: chronic obstructive pulmonary disease; OSAS: obstructive sleep apnea syndrome; PAE: pulmonary artery embolism. [1]: *n* (%); [2]: Mean (SD).

**Microbiological analysis**	***n*=**	**Positive findings**	**Negative findings**	***p*-value**
Number of patients[1]		270	102	
Age (years)[2]	372	65.0 (±13.9)	64.5 (± 14.1)	0.969
Male sex[1]	372	203 (75.2)	75 (73.5)	0.743
BMI (kg/m^2)[2]	266	28.4 (±5.5)	28.2 (± 6.7)	0.870
MI in the past/ CHD [1]	361	46 (17.6)	13 (13.1)	0.311
Vitium of aortic/mitral valve (grade 2/3)[1]	361	20 (7.6)	5 (5.1)	0.389
Heart failure ⩾ NYHA 3[1]	361	19 (7.3)	5 (5.1)	0.454
Atrial fibrillation[1]	361	31 (11.8)	14 (14.1)	0.554
Pacemaker[1]	361	10 (3.8)	2 (2.0)	0.396
Arterial hypertension[1]	361	135 (51.5)	45 (45.5)	0.304
Hyperlipidemia[1]	361	42 (16.0)	15 (15.2)	0.838
Diabetes mellitus[1]	361	48 (18.3)	15 (15.2)	0.480
Nicotine abuse (> 5py)[1]	361	66 (25.2)	25 (25.3)	0.990
Alcohol abuse[1]	361	22 (8.4)	3 (3.0)	0.074
Chronic renal failure KDIGO ⩾ stage 3[1]	369	24 (9.2)	10 (10.1)	0.785
Renal replacement therapy[1]	369	8 (3.0)	3 (2.9)	0.991
COPD ⩾ GOLD 2[1]	361	21 (8.0)	12 (12.1)	0.228
Bronchial asthma[1]	361	8 (3.1)	3 (3.0)	0.991
OSAS[1]	361	10 (3.8)	4 (4.0)	0.922

Apoplexy[1]	361	23 (8.8)	8 (8.1)	0.833
Thrombosis/PAE [1]	361	5 (1.9)	4 (4.0)	0.247
Malignant disease[1]	361	25 (9.5)	4 (4.0)	0.087
Peripheral arterial disease ⩾ stage 2[1]	361	10 (3.8)	4 (4.0)	0.922
Carotid artery stenosis[1]	361	9 (3.4)	4 (4.0)	0.783
Hypo- or hyperthyroidism[1]	361	19 (7.3)	8 (8.1)	0.790

Supplementary Table S5. Survival rates per SCAI stage of cardiogenic shock (A–E) for the overall cohort and separately for the MCS group and the non-MCS group. [1]: *n* (%).

	**Overall cohort**		**MCS**		**Non-MCS**	
**SCAI Stage of CS**	***n*=**	**Survivor**	***n*=**	**Survivor**	***n*=**	**Survivor**
SCAI Stage **A**[1]	13	13 (100.0)	/	/	13	13 (100.0)
SCAI Stage **B**[1]	17	16 (94.1)	/	/	17	16 (94.1)
SCAI Stage **C**[1]	90	68 (75.6)	/	/	90	68 (75.6)
SCAI Stage **D**[1]	141	73 (51.8)	42	26 (61.9)	99	47 (47.5)
SCAI Stage **E**[1]	111	33 (29.7)	73	23 (31.5)	38	10 (26.3)

Supplementary Table S6. Frequency of positive microbiological findings per SCAI stage of cardiogenic shock (A–E) in the overall cohort and in the MCS group and the non-MCS group. [1]: *n* (%).

	**Overall cohort**		**MCS**		**Non-MCS**	
**SCAI Stage of CS**	***n*=**	**Positive findings**	***n*=**	**Positive findings**	***n*=**	**Positive findings**
SCAI Stage **A**[1]	13	6 (46.2)	/	/	13	6 (46.2)
SCAI Stage **B**[1]	17	12 (70.6)	/	/	17	12 (70.6)
SCAI Stage **C**[1]	90	68 (75.6)	/	/	90	68 (75.6)
SCAI Stage **D**[1]	141	107 (75.9)	42	31 (73.8)	99	76 (76.7)
SCAI Stage **E**[1]	111	77 (69.4)	73	49 (67.1)	38	28 (73.7)
